# Аn affordability of statins therapy - comparative analysis between Ukraine and Bulgaria

**DOI:** 10.1186/s12913-019-4736-3

**Published:** 2019-11-27

**Authors:** Oksana Tkachova, Larysa Iakovlieva, Zornitsa Mitkova, Manoela Manova, Alexandra Savova, Guenka Petrova

**Affiliations:** 1 Department of Pharmacoeconomics, National University of Pharmacy, 4-Valentinivska str, Kharkiv, 61168 Ukraine; 2 Department of Organization and Economy of Pharmacy, Faculty of Pharmacy, Medical University-Sofia, 2-Dunavstr, 1000 Sofia, Bulgaria

**Keywords:** Statins, Prices per DDD, Affordability, Bulgaria, Ukraine

## Abstract

**Background:**

The statins are а developing group of cardiovascular medicines, widely used for dyslipidemia. As a whole statins consumption leads to reduction in cardiovascular events and death, and improves the disease control. The main study issue considers the differences in an affordability to lipid lowering medicines in the countries with the highest morbidity and mortally rate within and outside EU. The affordability has been researched by exploring the price differences and average wages.

**Methods:**

On total 7 international nonproprietary names and 19 dosage forms available on both markets are observed during 2013–2016. An average, minimum, and maximum retail prices per DDD, standard deviation (SD) has been calculated for all marketed dosage forms. A price ratio between the minimal and maximal price per DDD is estimated in order to evaluate their difference. Affordability of the treatment is determined as the number of working hours per month needed for patient to purchase medicines for a monthly therapy.

**Results:**

Large variations of price per DDD, SD and the average price exist between different dosage forms in both countries. The highest value of a price ratio is observed for 5 mg rosuvastatin in Bulgaria and 10 mg rosuvastatin in Ukraine.

The number of working hours needed to cover monthly therapy has increased during 2013–2016 in Ukraine. The most affordable is treatment with a generic atorvastatin in Bulgaria and generic rosuvastatin in Ukraine. The most expensive rosuvastatin in Bulgaria and atorvastatin in Ukraine are found as the least affordable for a monthly therapy.

**Conclusions:**

The decrease of prices for statins is not the only reason influencing patients’ affordability to therapy for statin therapy in Ukraine and Bulgaria. The difference in affordability in Ukraine and Bulgaria is affected mainly by the economic development in the country as well as wages variation.

## Background

Statins are recognized as one of the most important therapeutic groups for dyslipidemiatherapy. Evidences supporting reduction in LDL-Cafter statins utilization, especially second and third generation, continue to grow latest years [1].Systematic review and meta-analysis found that patients treated with statins demonstrated about 20 to 30% reduction in cardiovascular events and death [2].Mortality due to cardiovascular diseases (CVD) varies significantly within the European Union (EU). In Bulgaria it is up to 60% among men (the highest in EU), while in women, the burden ranges from 25% in Denmark to 70% in Bulgaria. Outside the EU, the CVD mortality in Europe also varies. In Ukraine it is found 59% among men, and 75% among women (the highest rate from countries outside of EU) [3, 4].A. large number of trials have demonstrated that statins significantly reduce the CV morbidity and mortality, as well as slow the progression of coronary atherosclerosis [5–7].The anging population and high morbidity suggest the high consumption of statins for treatment in dyslipidemia. Earlier studies show that treatment by statins has increased and expected to rise during the next years [8–10].The utilization of statins and prescribed dosages depends on patients’ stage of disease, body weight, concomitant diseases, complications etc. The wide variety of dosage forms allows a more precise dosing according to the individual needs of the patient and could also lead to price competition and improved affordability.

The adherence is defines as the degree to which the person’s behavior corresponds with the agreed recommendations, while compliance is the degree to which a patient correctly follows all recommendations [11].Therefore, the “compliance” suggests that the patient passively following the physician’s advices, whereas according to “adherence” definition the patient is part of the decision making process [12].Non-compliance and poor adherence could affect significantly results of the treatment. According to Guidelines for the management of dyslipidaemias, statins are among the most studied medicines in CVD prevention. Poor response to statins therapy is explained by a poor compliance, but may also be a result by a genetic variation [13]. Lots of medicines are still unaffordable to treatment in European Union (EU) between 2013 and 2015 [14]. Therefore the statins affordability is important to be studied.

The report of World health organization (WHO) reveals high medicines prices and unaffordable treatment in many countries by calculating thenumber of days’ wages required to purchase selected courses of treatment for acute or chronic conditionsand the daily wage of the lowest-paid unskilled government worker [15]**.** Where out-of-pocket paymentfor medicines is high, both high prices and low availability could worsen the compliance and diseases control [16]**. **Unaffordability to ambulatory treatment may increased direct and indirect costs of hospital therapy [14]. From the other side unaffordability means inequity in access to ambulatory therapy that deteriorates achievement of the primary goal of any health care system [17]**.**

Our interest in this study was prompted by the high rate of CVD morbidity and mortality, highutilization of statins as well as low incomes in both Ukraineand Bulgaria. The main study issue considers the differences in prices and affordability to lipid lowering medicines in the countries with the highest morbidity and mortally rate within and outside EU.

To explore the prices and affordability we have tested the differences between the average, minimal, and maximal retail prices per DDD, and have analysed the treatment affordability by exploring the price variations and average wages. Main research question was whether the prices decrease during the observed period and if the people can pay for their statins therapy.

## Methods

The analysis includes all international nonproprietary names (INNs) and dosage forms of statins (Anatomic therapeutic chemical (ATC) code C10AA) available on both markets. On total, 7 international nonproprietary names (INNs) and 19 dosage forms of statins are observed during 2013–2016.

### Price differences analysis

Data on prices of statins marketed in Bulgaria were collected from the price registry of the National council of pricing and reimbursement [18].To determine the available statins on the Ukrainian market a research system “Pharmstandard”of the company “Morion” is used [19].The study compares price per defined daily dose (DDD) of statins based on an approved retail prices of medicinal products in both countries.

An average, minimum, and maximum retail prices per DDD, as well as the standard deviation (SD) has been calculated for all marketed dosage forms.

In order to evaluate the price variability is estimated a ratio of differences between the minimum and maximum price per DDD using the formula:

$$ Price\ ratio= the\ highest\ price\  per\  DDD/ the\ lowest\ price\  per\  DDD $$

Pravastatin and pitavastatin are not marketed in a both countries. Therefore they are not included in a current price variation analysis.

### Affordability analysis

Affordability is determined as the number of working hours per month needed for patient to purchase medicines for monthly therapy. The methodology for affordability analysis of World Health Organization has been modified [15]. Average monthly wage was divided on the number of working hours per month thus determining the average cost of working hour. The cost of patients’ monthly therapy was calculated by multiplying the lowest or highest price per DDD on 30 days. Finally, the number of working hours or days that should be devoted to pay for monthly cost of therapy has been found.

1. Cost of monthly therapy = Lowest or highest price per DDD* 30
2. Wage per hour = ((Average monthly wage) / (22 working days) /(8 working hours))
3. Hours wage needed for a monthly therapy =Cost of monthly therapy / Wage per hour

All prices and wages were converted in € at the exchange rate of 1 BGN = 0.51 € in Bulgaria and 1 € equals 10.92 UAH in 2013, 1 € equals 17.12 UAH in 2014, 1 € equals 24.36 UAH in 2015 and 1 € equals 28.28 UAH in 2016 in Ukraine.

The data for an average monthly wage in Bulgaria was extracted from the National Statistical Institute database as follow 408.51; 441.15; 477.87 and 486.54 € for 2013, 2014, 2015 and 2016, respectively. According to officially published information by the National Bank of Ukraine, the amount of an average monthly wage is 299; 200; 172.2 and 183.4 € for 2013, 2014, 2015 and 2016 respectively.

The Friedman test and Wilcoxon signed-rank test were applied for statistical analysis of the data.

## Results

### Results of the price differences analysis

Large variations of price per DDD exist between different dosage forms in both countries. The minimal, maximal prices, and standard deviation (SD) per INN are listed in Table 1.

**Table 1:** The minimal, maximal price per DDD and SD during 2013–2016

	Min-max (SD) price per DDD – in Euro			
INN	2013	2014	2015	2016
*Ukraine*				
simvastatin	0.13–0.76 (0.23)	0.06–0.72 (0.24)	0.13–0.90 (0.31)	0.09–0.89 (0.30)
lovastatin^a^	0.36	0.27	0.22	0.22
fluvastatin^a^	0.65	0.43	0.23	
atorvastatin	0.14–1.78 (0.58)	0.09–1.50 (0.46)	0.06–1.92 (0.61)	0.02–1.86 (0.06)
rosuvastatin	0.04–0.82 (0.29)	0.04–0.70 (0.25)	0.03–0.57 (0.22)	0.04–0.49 (0.19)
*Bulgaria*				
simvastatin	0.06–0.79 (0.31)	0.06–0.79 (0.32)	0.06–0.79 (0.31)	0.06–0.79 (0.31)
lovastatin	0.26–0.28 (0.02)	0.26–0.28 (0.02)	0.28–0.28	0.28–0.51 (0.16)
fluvastatin	0.16–0.45 (0.20)	0.16–0.28 (0.08)	0.16–0.28 (0.08	0.16–0.52 (0.26)
atorvastatin	0.06–0.90 (0.32)	0.06–0.65 (0.20)	0.03–0.52 (0.16)	0.03–0.49 (0.15
rosuvastatin	0.13–1.28 (0.39)	0.10–1.28 (0.40)	0.10–1.28 (0.41)	0.10–1.28 (0.42)

^a^ Only one trademark is available on the market

The minimal price per DDD for simvastatin and fluvastatin in Bulgaria is lower than that in Ukraine. A reduction of the minimal prices of lovastatin and atorvastatin has been observed in Ukraine (Table 1).

SD shows huge variations of the INNs prices on both markets. The prices of rosuvastatin and simvastatin reveal the highest SD in Bulgaria, whereas atorvastatine - in Ukraine.

**Fig. 1:**
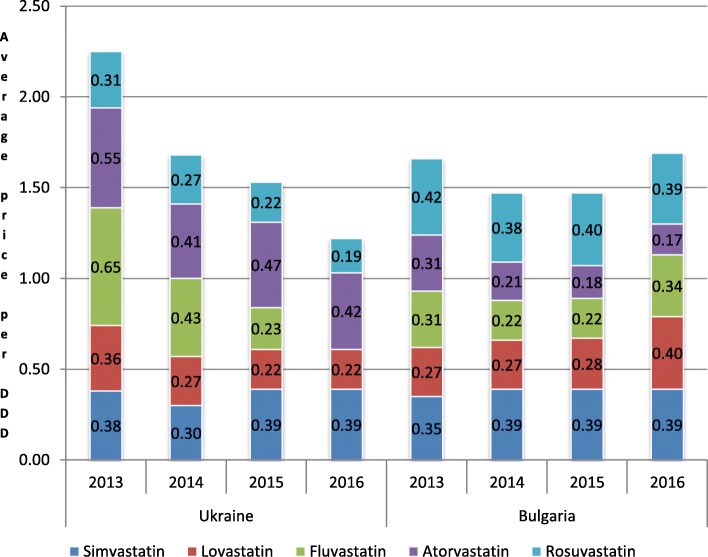
Average price per DDD in Ukraine and Bulgaria during 2013–2016

An average prices per DDD for INN are not similar in Bulgaria and Ukraine (Fig. 1). Lovastatin and fluvastatin are compared based on a their single price in Ukraine due to the lack of competitors.

Results from the Friedman test show that median values of the highest and lowest prices during the observed period are no statistically significant in both countries (*p* > 0.05) (Table 2).

**Table 2:** Results from Friedman test for comparison of highest and lowest prices during 2013–2016

Bulgaria						Ukraine					
Median value of highest prices per DDD		p	Median value of lowest prices per DDD		p	Median value of highest prices per DDD		p	Median value of lowest prices per DDD		p
2013	0,790	0,5606	2013	0,130	0,8731	2013	0,790	0,6471	2013	0,135	0,1312
2014	0,650		2014	0,100		2014	0,710		2014	0,0750	
2015	0,520		2015	0,100		2015	0,735		2015	0,0950	
2016	0,520		2016	0,100		2016	0,690		2016	0,0650	

The price ratio shows how many times the price of the most expensive exceed the price of the cheapest dosage form. It is found as a difference between the lowest and the highest price per DDD (Table 3).

**Table 3:** Price ratio between the most and least expensive dosage form

Price ratio					Price ratio				
INN	2013	2014	2015	2016	INN	2013	2014	2015	2016
*Ukraine*					*Bulgaria*				
Simvastatin 10 mg	2.30	4.00	5.63	4.24	Simvastatin 10 mg	4.15	4.15	4.15	4.15
Simvastatin 20 mg	2.83	3.58	3.60	3.06	Simvastatin 20 mg	8.21	8.21	8.21	8.58
Simvastatin 40 mg	3.00	5.33	3.62	5.78	Simvastatin 40 mg	5.61	9.19	9.19	9.19
Atorvastatin 10 mg	8.09	7.14	10.67	11.63	Atorvastatin 10 mg	8.67	6.77	6.05	5.69
Atorvastatin 20 mg	7.43	8.09	13.75	12.00	Atorvastatin 20 mg	10.97	7.21	7.29	7.29
Atorvastatin 40 mg	3.87	4.83	6.50	6.22	Atorvastatin 40 mg	5.58	3.29	7.63	7.63
Atorvastatin 80 mg	1.32	2.54	4.11	5.80	Atorvastatin 80 mg	2.70	1.54	1.54	4.19
Rosuvastatin 5 mg	3.57	3.50	3.56	4.80	Rosuvastatin 5 mg	8.78	11.30	11.30	11.73
Rosuvastatin 10 mg	18.75	16.50	14.25	16.33	Rosuvastatin 10 mg	5.97	6.10	5.74	7.95
Rosuvastatin 20 mg	4.31	11.75	13.67	12.00	Rosuvastatin 20 mg	4.76	6.23	6.23	6.00
Rosuvastatin 40 mg	2.00	5.50	3.43	4.20	Rosuvastatin 40 mg	2.37	1.22		

The highest value of price ratio is observed for 5 mg rosuvastatin in Bulgaria and 10 mg rosuvastatin in Ukraine.

### Results from the affordability analysis

The affordability section researches relationship between monthly treatment costs and monthly patients’ wages, showing how it differs during the observed period of time. Table 4 illustrates the number of working hoursneeded for a payment of monthly statins therapy.

**Table 4:** Number of working hours needed for the monthly treatment course

	Number of working hours needed to cover the low and-high cost therapy			
INN	2013	2014	2015	2016
*Ukraine*				
simvastatin	2.30–13.42	1.54–18.90	3.99–27.60	2.59–25.62
lovastatin^a^	6.36	7.15	6.75	6.33
fluvastatin^a^	11.48	11.33	7.05	–
atorvastatin	2.47–31.43	2.46–39.63	1.84–58.87	0.58–53.55
rosuvastatin	0.71–14.48	1.06–18.53	0.9–17.48	0.86–14.11
pitavastatin	6.18–15.36	8.92–21.07	11.34–24.84	10.08–23.61
*Bulgaria*				
simvastatin	0.73–10.24	0.68–9.48	0.62–8.75	0.61–8.60
lovastatin	3.28–3.68	3.07–3.41	2.84–3.15	3.09–5.57
fluvastatin	2.09–5.82	1.94–3.34	1.79–3.08	1.76–5.69
atorvastatin	0.79–11.66	0.67–7.74	0.36–5.75	0.35–5.31
rosuvastatin	1.73–16.59	1.21–15.36	1.12–14.18	1.06–13.39
pravastatin	6.37–7.03	5.90–6.51	5.45–6.01	5.35–5.91

^a^ Only one product is available on the market

The study found a large differences in the affordability between the highe stand the lowest-priced statins. The most affordable is treatment with a generic Atorvastatin (0.79–0.35 h wage for the lowest priced product in 2013–2016, respectively) in Bulgaria, and generic rosuvastatin in Ukraine (0.71–0.86 h wage for the lowest priced product in 2013–2016, respectively). The less affordable is treatment with most expensive rosuvastatin in Bulgaria (16.59–13.53 h wage in 2013–2016, respectively) and atorvastatin in Ukraine (31.43–53.55 h wage in 2013–2016, respectively). Number of working hours needed to pay for generic Simvastatin is between 2.30–2.59 € in Ukraine and 0.73–0.61 €in Bulgaria during 2013–2016. The highest priced Atorvastatin is between 31.43–53.55 € in Ukraine and 11.66–5.31 € in Bulgaria during 2013–2016.

Results of the Wilcoxon signed- rank test reveal that there are no statistically significant differences in affordability, neither comparing Bulgaria with Ukraine, nor comparing the affordability during the years (*p* > 0.05).

## Discussion

The availability of statins on the national markets is almost equal and the approved INNs and dosage forms are almost the same. Thus we can confirm that patients’ physical access to variety of INNs and dosage forms of statins has been granted.

The prices of available statins reveal significant variation. The average price per DDD decreased during 2013–2016, except fluvastatin and lovastatin prices in Bulgaria. The rising of latter products prices is probably affected by other products withdrawal from Bulgarian market. Thus we can consider that we found evidences for the prices decrease meaning that the medicines became more affordable for the population.

Major differences between the lowest and the highest priced products are observed. The price ratio varies from 1.23 to 18.75 in Ukraine and 1.22–11.73 in Bulgaria. This could be explained by the discrepancies in a medicines prices and lack of officially stated generic medicines policy. It also pointed out that there is a risk for patients’ access to low priced medicines.

The price ratio of generic to originator medicines is found between 0.34 and 0.98 in China hospitals during 2006–2011. The potential savings are about 65% if patients switch form originator to generic medicines [20].The generics lead to cost reduction and also improve the access to medicines in the countries with increasing population and shrinking resources [21].

Analysis of medicines availability in 36 countries found that the average public sector availability of generic medicines ranged from 29.4 to 54.4% across WHO regions. The policy options and alternative financing mechanisms could increase availability, reduce the prices, and to improve medicines affordability [22].

49.9% is the median price differences between generic and originator medicines in South Africa. Of all analyzed generic medicines, 67.5% were more than 40% cheaper per defined daily dose (DDD) per month, than the branded [23]. Prices of generic and originator antiepileptic drugs in India reveal large price variations [24], and the main reason is the established pricing policy [25].

We did not explore the price control systems in both countries. A reference price system based on the lowest price out of 17 reference countries is implemented in Bulgaria. National Council on Pricing and Reimbursement approves medicines prices and defines reimbursement level in the range 25–100%.The approved prices of the reimbursed medicinal products on a retail level are official for the whole country. The variation of prices is a result of the high number of trade names. The medicines approved for reimbursement are listed in Positive Drug List divided into 3 Annexes according to the payment institution. Annex I includes medicines for ambulatory care paid by the Health Insurance Fund. The reimbursement level for statins is 25% and as it pointed out in the methodology the prices was gathered from the PDL so 25% of the observed prices is reimbursed. Annexes II and III includes medicines for hospital care and socially important diseases (AIDS, infectious diseases, vaccines etc.), paid by the Health Insurance Fund and Ministry of Health, respectively.

In March 2017, the Cabinet of Ministers of Ukraine and the Ministry of Health of Ukraine have adopted a number of bylaws representing a major overhaul of the regulatory framework for pricing and the reimbursement of pharmaceuticals in Ukraine [26]. On 1 April 2017, reimbursement was launched with respect to 21 INNs of pharmaceuticals for treating cardiovascular diseases, type II diabetes and asthma. A medicine can be included in the Reimbursement Register if its price does not exceed the maximum wholesale price. The maximum wholesale price is calculated as a median of the prices for the respective pharmaceuticals in the reference countries (Poland, Slovakia, Latvia, Hungary and Czech Republic), based on the daily defined dose established by the World Health Organization [27].Now more than 13 million people (12 million people suffering from CVD, 1 million - type 2 diabetics and 210 thousand people withasthma) have the right to claim partial/full compensation for the cost of the medicines in pharmacies. Since the beginning of the program, about 2 million prescriptions have been written out. As a whole, the regulatory measures are expected to improve the affordability in a future but the first steps are encouraging.

In general, we can say that both countries are trying to improve the affordability to lipid lowering medicines either via price control or via national programs [28].

Other studies found that the cost of therapy, increased number of prescribed medicines, and patients’ diseases are main factors affected patients‘adherence to therapy [29, 30]. In Ukraine is establishing poor adherence to therapy, whereas comparatively high level is reported for Bulgaria (between 50 and 92%). The limitation of this study is a lack of adherence data and more specifically findings revealing the clear linkage between adherence and medicines reimbursement in compared countries. Because of that it is not completely commented. Further studies needs to be done to explore the relationship between the affordability and adherence to therapy.

There is a reverse relationship between prices of medicines and their utilization. The large differences between generic and branded medicines could influence patients’ affordability to treatment and results of the therapy.

The economic growth or decline could play a crucial role in affordability to treatment. The annual GDP and variation in the average annual salary are the main economic factors influencing medicines utilization. In Bulgaria the average wage slightly increases from 408.51 € in 2013, 441.15 € in 2014, 477.87 € in 2015, to 486.54 € in 2016. On the contrary in Ukraine the average wage mostly decreases – 299 € in 2013, 200 € in 2014, 172,2 € in 2015, 183,4 € in 2016. This fact significantly affected the study results.

GDP per capita is 5400 € in 2013 in Bulgaria, and it grows to 6000 € in 2016 [31].A slow economic development and slight increase of average annual wage ensures better affordability to treatment in Bulgaria. For example, GDP per capita is 2186 U.S. dollar in 2016 in Ukraine [32].

On national level we could comment that more working hours are needed for statin therapy in Ukraine. We have found a reduction in the mean price per DDD (except simvastatin), minimal and maximal prices per DDD (except the maximal price per DDD of Simvastatin and Atorvastatin) but the number of working hours needed for monthly therapy increased as a result of the drop down incomes in Ukraine.

The utilization of statins increased during 2005–2007 in Bulgaria [8].The prices significantly have decreased and the utilization simultaneously has increased in 2009–2013. The high number of generics was approved on Bulgarian market thus affecting prices and affordability [33].The number of necessary working hour’s decreases, due to the risen wages. It confirms the improvement in affordability in Bulgaria.

Patients have to pay between 5 and 7 days’ wages to buy 1 month’s treatment by amlodipine and simvastatin in Malaysia. Patients have to work 3 days to purchase generic simvastatin. A large part of the population would not be able to pay for their medicines [34]. WHO study found that from 2.0 to 8.3 day’s are needed to purchase 1 month treatment with cardiovascular medicines. Treatment by originator was 4.2 times as much as buying the lowest-priced generic. One month of chronic hypertension treatment was 1.8 day’s wages [35].Similar survey in Republic of Moldova found that 1.85 working days for lowest income people are needed to purchase 1 month of cardiovascular disease treatment in 2006 and 0.84 days in 2013. Introduction of mandatory health insurance increased the affordability, as well as the raising household incomes [36]. We can comment that according to our results the affordability is much better in Bulgaria and Ukraine comparison with above studies but if generic alternatives are used. There is no explicitly stated threshold how many working hours are necessary for being able to afford all necessary medicines, but in general we can say that low priced medicines which require 0,7 h of work could be considered as affordable [14]. Therefore introducing generic medicines policy will increase affordability. Our study confirms that in the countries with better economic results the affordability is better. Overall, the encouraging generic policy on a country level and a lower out -of pocket payment would improve the patients’ affordability.

Other factors as companies’ policy could affected the price differences and explain some contradictions among less and high costly molecules.

## Conclusion

The decrease of prices for statins is not the only reason influencing patients’ affordability to therapy for statin therapy in Ukraine and Bulgaria. The difference in affordability in Ukraine and Bulgaria is affected mainly by the economic development in the country as well as wages variation.

## Data Availability

Administrative permission of the Morion company was obtained for access to data on research and analysis of medicines on the pharmaceutical market of Ukraine. Data for Bulgaria have been extracted from the official public registers of National Council on Prices and Reimbursement of Medicinal Products, available at: http://ncpr.bg/en, as well as National statistical institute, available at: https://www.nsi.bg/en Please contact the corresponding author Oksana Tkachova, tkachevaov@gmail.com for data requests.

## Abbreviations

CVD: Cardiovascular diseases
DDD: Defined daily dose
EU: European Union
GDP: Gross domestic product
INNs: International nonproprietary names
LPG: Lowest priced generic
OB: Originator brand
SD: Standard deviation
WHO: World Health Organization
